# Providing Hope or Assigning Blame? Healthism in Print Media Portrayals of Dementia Risk and Responsibility

**DOI:** 10.1111/1467-9566.70115

**Published:** 2025-11-11

**Authors:** Felicity Slocombe, Elizabeth Peel, Alison Pilnick, Saul Albert

**Affiliations:** ^1^ Faculty of Health and Social Care Department of Applied Dementia Studies University of Bradford Bradford UK; ^2^ Centre for Research in Communication and Culture School of Social Sciences and Humanities Loughborough University Loughborough UK; ^3^ Faculty of Health and Education, School of Nursing and Public Health Manchester Metropolitan University Manchester UK

**Keywords:** blame discourses, dementia, focus groups, healthism, media representations, responsibility, risk factors, stigma, thematic discourse analysis

## Abstract

Media representations often imply that dementia is preventable through lifestyle choices, potentially blaming individuals for their condition. Crawford's ideology of healthism outlines this form of responsibilisation. Our thematic discourse analysis of focus group discussions demonstrates how different stakeholder groups interpret print media messages differently, with prevention messaging that promotes health behaviours among the general population, simultaneously felt as stigmatising those already affected by dementia. Participant discussions almost exclusively focused on individual‐level risk factors (e.g., diet and genetics) rather than population‐level modifiable risks (e.g., air pollution and education), reflecting current print media and policy framings. It is worth noting that while participants saw their own/their loved one's dementia as unpreventable, they viewed other cases, especially vascular dementia, as preventable through health choices. Our analysis shows how healthist ideals especially stigmatise specific dementia diagnoses, highlighting where healthist messaging may be particularly harmful. We recommend that media outlets and policymakers emphasise population‐level interventions alongside individual actions, and avoid language implying personal responsibility for developing dementia. Our analysis demonstrates how Crawford's healthism is relevant for understanding media representations of dementia, as well as highlighting some much‐needed changes.

## 1 Introduction

In the United Kingdom, around one million people live with dementia, and there are more than 200 different types, including Alzheimer's disease, vascular dementia, Lewy body dementia, and frontotemporal dementia (Dementia UK 2023). Approximately 7.5% of diagnoses are for young‐onset forms of dementia, where symptoms begin before the age of 65 (Dementia UK 2023). Dementia is not a homogenous condition and symptomatic experiences and impacts on day‐to‐day life vary (Rockwood et al. 2014). It can impact communication, behaviour, personality, memory and mobility, among other things (Dementia UK 2023). For people experiencing young‐onset dementia, there can be additional challenges as they may still be working and/or have dependent children/parents (Harris and Keady 2009). However, increasing age is still the single largest risk factor for dementia (Alzheimer's Society 2025). All types of dementia are progressive, and although there have been recent advances in biomedical treatments, side effects and costs are barriers to use (e.g., Hafiz et al. 2023).

Daly (2023) argues that Crawford's (1980) discussion of healthism, an ideology which emphasises individual responsibility and moral obligation for health maintenance, still underpins contemporary public discourse around dementia. As medical searches for cures continue with limited success (Whitehouse and George 2014), the focus of dementia research has shifted towards prevention and risk reduction. Recently Livingston et al. (2024) identified 14 potentially modifiable risk factors^1^ for dementia (see also Livingston et al. 2017, 2020). This shift of emphasis to modifiable risk factors, however, also risks discursively reframing dementia as resulting from a failure to lead a healthy lifestyle (Marhánková 2022). This focus on prevention as an individual's responsibility is a growing discourse within the biomedical view of dementia, and disease more widely (Latimer 2018; Petersen and Schicktanz 2021). Lawless et al. (2018b) demonstrated how dementia charities across seven nations reinforce this perspective through the content of their websites. These webpages framed individuals as personally responsible for preventing potential dementia, promoting actions such as ‘keep your brain active’, ‘look after your heart’, and ‘eat healthily’. In German newspapers, Daube et al. (2023) found responsibility was prominently framed in almost all articles. Risk factors were mentioned in over 52% of articles, with the onus being on the individual to manage that risk within 89.5% of these articles. In the United Kingdom, Peel (2014) argued that newspapers' lexical choices such as ‘ward off’, ‘reduce’, ‘avoid’ and ‘prevent’ present dementia as avoidable through personal actions. Similarly, Putland and Brookes (2024) found individual responsibility to be a wider pattern in their scoping review of dementia stigma and language research, particularly in newspapers.

This healthist reframing of dementia plays into the dubious claims of the emerging ‘brain training’ industry (L. Nguyen et al. 2022) and the imperative to cognitively ‘use it or lose it’ (e.g., Allen 2021). Without explicitly mentioning dementia, Crawford's work does allude to conditions that impact identity at the boundaries of the self and the ‘unhealthy other’. For example, Crawford's (1994: 1355) study of HIV/AIDS health culture describes how ‘to a certain extent, all serious affliction is an inscription of otherness’, and that conditions impacting identity can bring about ‘psychic disintegration’ with a range of impacts, projecting an ‘*internal* other’ (emphasis added), aligning with entrenched stigma related to (loss of) identity in a dementia context (T. Nguyen and Li 2020). In this paper, we build on the foundations of Crawford's work and apply healthism directly to the context of dementia.

Given the United Kingdom's ageing population, cost‐of‐living crises and public service cuts (Barton et al. 2024; Kenyon 2023; Ogden and Phillips 2024), healthism serves a political function. Crawford (1980) himself, and Daly (2023) argue that healthism frames health as non‐political, by making individuals exclusively accountable for their own health outcomes, thus framing health as falling outside the responsibility of politicians. Researchers studying dementia risk factors consistently argue that future wide‐scale reduction of dementia cases requires individual *and* policy interventions (Daly 2023; Livingston et al. 2017, 2020, 2024; Walsh et al. 2022; Wilson and Anstey 2024), yet these critical recommendations rarely receive media attention (Marhánková 2022). Dementia is not the exception, and healthist discourses are visible in other conditions where individual health behaviours are seen to play a part in causation (e.g., lung cancer: Tien et al. 2024). Petersen and Schicktanz (2021) and Wilson and Anstey (2024) have analysed how the nuance is lost within newspapers in favour of an oversimplified message about individual modifiable risk factors for dementia, that does not consider how different risk factors interact. For example, impaired mental and physical health affect an ability to engage in exercise or social activities (Intzandt et al. 2015). Low and Purwaningrum (2020) argue that oversimplified media representations can create stigmatising perceptions of dementia with serious consequences: people with dementia may withdraw from social contact (Milne and Peet 2008), hesitate to seek support and diagnosis, and experience damaged well‐being (Iliffe et al. 2005). Although dementia undoubtedly has real impacts on people's lives regardless of societal representation (Bartlett et al. 2017), previous work with people with dementia suggests that these dominant narratives can worsen stigma and negative experiences (Alzheimer’s Society 2024; Dementia Engagement and Empowerment Project, DEEP 2014). In this paper, we draw on T. Nguyen and Li’s (2020) distinctions between public‐stigma and self‐stigma:

Table 1 illustrates how stereotypes, prejudices and discrimination can impact on societal and individual levels. Public‐stigma and self‐stigma contribute to a politics of responsibility by framing dementia not solely as a medical condition but as a personal failure not to prevent it, which Latimer (2018) labels the ‘new stigma’. Public‐stigma can position people with dementia as burdens or incompetent, while self‐stigma can result in internalising these views, aligning with the healthism narrative that individuals are responsible for their cognitive decline, resulting in possible feelings of shame or guilt. Understanding stigma through the lens of healthism can help us appreciate how societal discourses can not only marginalise people with dementia but also assign them undue responsibility for their condition.

**TABLE 1:** Matrix of public‐stigma and self‐stigma.^a^

Level/Aspect	Type of stigma	
	Public‐stigma	Self‐stigma
Stereotype (belief)	Negative belief about a group (e.g., dangerousness, incompetence)	Negative belief about the self (e.g., weakness, incompetence)
Prejudice (emotion)	Agreement with the belief and/or negative emotional reaction (e.g., anger, fear)	Agreement with the belief, negative emotional reaction to themselves (e.g., upset, self‐distrust)
Discrimination (behaviour)	Behavioural manifestation of prejudice (e.g., avoidance, help‐withholding)	Behavioural response to prejudice (e.g., self‐isolation)

^a^ Quoted from T. Nguyen and Li (2020, 150) who adapted the definitions from tables in Corrigan et al. (2005, 182) and Corrigan and Watson (2002, 16).

Crawford's (1994) stigma‐related work of the 1990s has parallels with contemporary dementia media discourses. When using the example of HIV/AIDS, Crawford (1994) describes how people were ‘othered’, viewed as unhealthy and lacking self‐control and how HIV/AIDS was branded an ‘epidemic’, just as dementia has been (Peel 2014). Crawford describes how the divide between the healthy self and unhealthy other can be seen in language through ‘the tacit assumptions so frequently heard in everyday conversations: “He has lung cancer.”/“Is he a smoker?” or… “He has AIDS.”/“Is he gay?”’ (1994: 1356). In dementia this could look like: ‘They have dementia.’/‘Did they lead a healthy lifestyle?’, with this rhetoric observable in government policy (Department of Health 2015; Office for Health Improvements and Disparities 2022).

Where many studies of dementia representations have focused on the interpretative analysis of media texts and framings (e.g., Daube et al. 2023; O'Malley et al. 2022; Van Gorp and Vercruysse 2012), our study uses Crawford's healthism to centre the perspectives of diverse stakeholders. Our approach builds on a smaller number of related studies. Peel's (2014) thematic discourse analysis of newspapers and interviews with family carers of people with dementia found that individual responsibility discourses appeared in the media, but not in carers' talk. Similarly, Yemm et al.’s (2023) study of perceptions of cognitive impairment in participants with different experiential and professional identities noted that participants did not spontaneously link lifestyle and cognition. Lawless et al. (2018a) studied comments on a Facebook page created to discuss dementia risk and prevention and, here they found frequent discussion of risk and accountability amongst users. Putland's (2022) study of how people with dementia and familial carers perceive dementia media representations found how seemingly positive or educational depictions of dementia can still create damaging representations of people living with the condition. By gathering perspectives from people with dementia, carers of people with dementia, and those less personally connected to dementia, our study shows, firstly, how Crawford's healthism manifests in contemporary media representations; and secondly how a range of stakeholders reproduce, challenge and resist these healthist ideologies. Through focus groups with diverse stakeholders, we extend Crawford's healthism to media representations of dementia, exploring specifically how healthism shapes media discourses about dementia risk and prevention, and how participants respond to them. Through our analysis of focus groups participants' perspectives, we ask: how does Crawford's healthism help us understand the way people with differing identities speak about and engage with media discourses of dementia?

## 2 Analytical Approach and Method

### 2.1 Analytical Approach

The analysis focused on video recordings and transcripts from three focus groups with three separate stakeholder groups: people with dementia, carers of people with dementia, and people with less personal connection to dementia. Our analytical approach was a thematic discourse analysis. This involved combining reflexive thematic analysis with discursive psychology, using a social constructionist framework (Taylor and Ussher 2001). Reflexive thematic analysis was used to identify patterns within talk, centring researcher subjectivity in the construction of themes (Braun and Clarke 2006; Braun et al. 2019). We constructed the themes from multiple rounds of transcript data coding (Braun and Clarke 2019) whilst also applying insights from discursive psychology (e.g., Edwards and Potter 1992). Our approach to the subsequent thematic analysis was deductive in that there was an existing interest in coding the construction of discourses related to healthism. Discursive psychology was used to examine psychological concepts and discursive devices within these themes (Wiggins 2017). Discursive psychology is a type of discourse analysis which has been applied to study both spoken and written language (Potter and Wetherell 1987; Edwards and Potter 1992). It does not comment on the reality or factuality of discourse but instead focuses on how participants construct versions of reality and the social actions that these versions of reality achieve in their specific interactional context (Potter 2020; Wiggins 2017). Discursive devices, such as hedging, extreme case formulations, and object‐ and subject‐side constructions (see Table 2) are essential tools in discursive psychology that allow investigation of psychological and social actions (Wiggins 2017). This combined approach allowed exploration of the ideological implications of dementia media discourses, and the discursive strategies participants use in responding to them, complementing the focus on healthism. The themes are ‘the what’ and the discursive psychology is ‘the how’ in relation to participants' talk within the topics of interest. In this paper we examine how discursive devices are used within the themes we identified in our analysis.

**TABLE 2:** Discursive devices used in the thematic discourse analysis.

Discursive device	Description	Effect	Examples from dataset
Hedging: See Wiggins (2017)	Talk marked as conditional or tentative	May help to manage the speaker's accountability by avoiding saying you are certain about something that could later be challenged by another interlocuter	‘I'm not sure it is the magic bullet really’
Extreme case formulations: See Pomerantz (1986)	Presenting something in an extreme	They can legitimise a claim or present something as objective, rather than a belief	‘Total false information’
Object‐side tellings: See Edwards (2007) and Potter (2020)	Talk built as factual or objective	This constructs the talk as separate from the interlocuter	‘It keeps your brain active, but it doesn't prevent it’
Subject‐side tellings: See Potter (2020)	Talk built as reflecting own experiences or opinions	People can therefore be accountable for knowing or believing the content of the talk	‘I don't honestly think there's anything you can do that would stop you getting a dementia’

### 2.2 Methods

Ethical approval for the study was obtained from the Loughborough University Ethics Review Sub‐Committee. Participants were recruited via their participation in an earlier online survey on the same topic of media representations of dementia, through advertising on Twitter (now X), and through existing mailing lists, mainly in charity/activism/academic spaces. Participation was open to people with any or no relationship to dementia. Thirteen people participated (see Table 3). Participants chose whether to be anonymous or identifiable in any research outputs in recognition that people affected by dementia have historically been excluded from research (Brooks et al. 2017) and may want to be recognised for their contribution (Giordano et al. 2007). All participants self‐selected to participate and indicated they had capacity to consent when they completed the informed consent form. Capacity to consent was also monitored throughout communications via email/online meetings as part of data collection. Based on information on their relationship to dementia sourced from a participant survey, participants were assigned to one of the three separate focus groups. This was to allow multiple viewpoints to be explored across the focus groups and to represent a variety of stakeholders in the research, especially people with dementia. As knowledge of dementia varies between people with different relationships to dementia will likely exist (Blendon et al. 2012), having separate groups based on similar characteristics aimed to help build common ground and a shared sense of identity between participants (Hydén and Bülow 2003). We do not advocate for the point of view of a specific stakeholder group but instead seek to demonstrate how an individual's frame of reference may impact their perceptions of dementia discourses.

**TABLE 3:** Participant information.

Participant name or pseudonym	Age; sex	Relationship(s) to dementia	Country of residence
Participants with less personal connection to dementia			
David	56; male	Grandparent had dementia; mother‐in‐law has dementia	England
Emma	55; female	General practitioner (GP) with special interest in older people; works in an education and leadership role around dementia	England
Henry	60; male	Grandmother and mother‐in‐law had dementia; suspects mother has dementia; occupation as an academic with experience of social/psychological dementia research	England
Participants who provide(d) care for a family member with dementia			
Gerry	68; male	Cares for wife who has young‐onset Alzheimer's disease	England
Michelle	65; female	Cares for husband who has young‐onset Lewy body dementia	England
Geoff	72; male	Cares for wife who has young‐onset Alzheimer's disease	England
Violet	60; female	Cared for mother‐in‐law who had Alzheimer's disease; volunteers with a dementia charity	England
Hannah	36; female	Cared for father who has Lewy body dementia; grandfather had Alzheimer's disease; occupation as an academic researching dementia from a cognitive psychology perspective	Republic of Ireland (originally from USA)
Gemma	47; female	Cared for mother‐in‐law; occupation as a community occupational therapist (OT) working in an NHS memory assessment and post‐diagnostic service. Facilitates a DEEP (dementia engagement and empowerment project) network group as part of this role	Scotland
Participants with dementia^a^			
Eleanor	62; female	Has Alzheimer's disease (diagnosed at 57); cared for parent	Northern Ireland
Julie	59; female	Has Alzheimer's disease (diagnosed at 54); worked with people with dementia as a nurse and social worker; cared for parent	England
Anita	54; female	Has mixed dementia; Alzheimer's disease and vascular dementia (diagnosed at 51); worked with people with dementia as a social worker; cared for friend	England
Martin	64; male	Has posterior cortical atrophy (diagnosed at 58); worked with people with dementia as a personal carer	Scotland

^a^ All participants with a dementia diagnosis have young‐onset dementia (symptoms began before the age of 65). This is likely to have impacted their experiences. Eleanor, Julie and Martin already knew one another prior to this research.

Four people participated in the ‘people with dementia’ focus group, six people participated in the ‘people who provide(d) care for a family member with dementia’ focus group and three people participated in the ‘people with less personal connection to dementia’ focus group (see Table 3). In total, 10 people with dementia participated in, or are referred to in the focus groups, because those in the carers' group all drew on the experiences of those they cared for. Young‐onset forms of dementia affected seven of these people: four participants living with dementia, and three people who participants cared for. The focus group questions and newspaper excerpts for discussion (see Supporting Information S1) were posted to participants in advance of their focus group, enabling advance preparation. Most participants (11/13) attended an initial meeting for their focus group, allowing them to meet one another and the facilitator (FS) and raise any questions. The three initial meetings and three focus groups were conducted online via Zoom (due to participant preferences) in Autumn 2022. Participants were asked a series of questions relating to media representations of dementia including: their most important source of news information; their beliefs around media messages impacting concern about developing dementia; their beliefs around reducing dementia risk through health and lifestyle changes; and whether they have changed their health behaviours because of media messaging. We also discussed excerpts from two newspapers articles about dementia (see Supporting Information S1), asking participants about their views on these articles, how they presented dementia and how they could impact wider perceptions of dementia. Across the three focus groups, we recorded 5.7 h of data (average length = 1 h 56 m, range = 1 h 50 m–2 h 04 m). All participants were White and most identified as heterosexual, with one participant identifying as bisexual, and one opting not to answer. Seven participants identified as Christian, four as having no religion, and two as Atheist.

Prior to the focus groups, consultation about focus group question suitability was undertaken with a person living with dementia who had significant prior research experience, and some minor amendments were made. For the focus groups themselves, it proved difficult to recruit people with no personal connection to dementia at all, perhaps due to recruitment being in charity/activism/academic spaces and because participation is more likely if people have an existing interest in the research topic (Stunkel and Grady 2011). This could also reflect the pervasiveness of dementia, as it is estimated one in two people in the United Kingdom will be affected in their lifetime (Besley et al. 2023). The participants with less personal connection to dementia still all either knew or had known people with dementia, or worked with people with dementia, making it difficult within this study to comment upon how people with no connection to dementia at all may interpret dementia media representations. After the focus groups, participants were able to read a copy of their group's transcript to ensure it accurately represented what they had said. Participants were also sent a findings summary blog.

## 3 Analysis

Our analysis focuses on two themes organised around participants' discussion of discourses relating to healthism in dementia media representations: (1) dementia (un)preventability, and (2) demonstrating dementia diligence. The prevalence of those with experience of young‐onset dementia becomes salient in numerous points of our analysis. Quotes from participants are used to illustrate the themes and show common discursive devices used by participants. Transcription conventions are used within excerpts to highlight aspects of talk, such as emphasis (see Jefferson 2004).

### 3.1 Dementia (Un)Preventability

This theme presents two sides of a preventability discourse in media representations of dementia. ‘Dementia as Unpreventable’ represents how participants present their *experiences* in comparison to media representations of modifying dementia risk, and ‘Dementia as Preventable’ represents how participants present the *implications* of media representations of dementia risk.

#### 3.1.1 Dementia as Unpreventable

For those personally affected by dementia, media messages around the possibility of reducing dementia risk, or delaying onset of dementia by engaging in health behaviours that ‘keep your brain active’ were treated as personally and generally inapplicable. Participants built narratives that they, or people they care(d) for, did everything recommended by research and subsequently in the media: they kept a healthy lifestyle, kept their brains ‘active’, and did not drink alcohol or smoke, but this did not prevent dementia for them. These narratives arose spontaneously before focus group questions related to blame discourses were asked, in contrast to findings of related research from Yemm et al. (2023). Below, Eleanor, a person with dementia, exemplifies the unpreventability theme, describing her own behaviours in relation to an excerpt of a newspaper article (Allen 2021) discussed in the focus groups (see Supporting Information S1). The facilitator (FS) has just read the excerpt aloud, which quotes research findings that playing board games, doing puzzles, card games, reading and writing letters can delay dementia by up to 5 years, Eleanor's response immediately follows:

Well like I play board games. I don't do card games. I do do puzzles. I like reading, and I do‐ I would have wrote letters ((laughs)) but that didn't work for me. ((laughs)).

Eleanor (person with dementia)

Eleanor's well‐prefaced response (Heritage 2015) anticipates her disalignment with the previous discussion, describing from a personal perspective, how the risk‐reduction described in the article ‘didn't work for me’. This personalised challenge to the preventability discourse was used by all participants with more personal connection to dementia (carers and people with dementia), through describing media messages of risk reduction as ‘not applicable’ (Michelle, carer), ‘hard to reconcile’ (Hannah, ex‐carer and academic), or ‘unrelatable’ (Anita, person with dementia). By doing this, participants highlight a discrepancy between following preventive advice, and the fact that they, or the person they care(d) for, still developed dementia. The high percentage of participants affected by young‐onset dementia could further explain why dementia risk reduction media messaging was presented as personally inapplicable, as partaking in recommended risk reduction strategies did not only fail to prevent dementia but also failed to delay onset until older age. Through challenging blame discourses, participants reduce potential threats to their identity of being or caring for a person who did not strive for utmost health, as implied in simplified healthist dementia risk representations. This finding is contrary to previous research a decade ago which found carers rarely acknowledged discourses of individual responsibility, despite their media presence (Peel 2014). This could suggest that there has been an increase in the presence of individual responsibility discourses around dementia, and/or carers' (and people with dementia's) awareness of these.

Conversely, participants with less personal connection to dementia did not draw on personal narratives to discuss inapplicability of risk reduction media messages, as this was not part of their frame of reference. Instead, they displayed a view that this same article excerpt could be reassuring and communicate a hopeful message that you can be in control of your own dementia risk. This echoes previous findings that media information that raises awareness of risk factors can nevertheless create damaging portrayals of people with dementia that does not reduce dementia stigma (Peel 2014; Putland 2022; Van Gorp and Vercruysse 2012). A participant in the carers' focus group explicitly recognised this dichotomy, by saying ‘we're all quite like involved in this, and… I'm guessing maybe we read these [the article excerpts] different to how‐just the man on the street might’ (Violet, ex‐carer). The presence and awareness of personal experience influencing perceptions of dementia risk messaging underlines the importance of examining the views of people impacted differently by dementia (Miranda‐Castillo et al. 2013).

Participants with less personal connection also questioned the legitimacy of media reporting, labelling it as potentially sensationalised and simplistic. However, participants such as Henry (academic) recognised that reporting on risk reduction research could provide hope of avoiding dementia for people currently unaffected, with this described as fitting established ‘commonsense’ understandings of keeping your mind active to delay dementia. Although Henry acknowledged that risk reduction media messaging is not ‘aimed at’, and does not reduce stigma for, people already affected. In presenting media discourses of dementia risk as inapplicable on a personal level, participants most personally affected by dementia refuted possible links between themselves and personal responsibility for dementia, creating discursive distance from implication in any causal attribution. This was expanded to dementia on a general level in some instances, and particularly by participants who have dementia, such as Anita and Eleanor below:

it‐ it [the things Allen (2021) recommends] keeps your brain active, but it doesn't prevent it [dementia]. And it doesn’t stave it off at all: so it was total false information.

Anita (person with dementia)

I think by: keeping active, by keeping involved, by stimulating, y’know you can sort of aid your journey through the dementia diagnosis, but I don't honestly think there's anything you can do that would stop you, getting a dementia.

Eleanor (person with dementia)

Anita and Eleanor both use extreme case formulations (ECFs, Pomerantz 1986) in distancing dementia from being preventable by keeping your brain active: ‘at all’, ‘total false information’, and (isn't) ‘anything you can do’. These ECFs counter the messages of health behaviours being able to reduce risk, or delay onset of dementia, presenting the article's contents as being completely wrong. Eleanor argues that behaviours that keep your brain active *may* help with dementia symptoms but will not stop you getting dementia, a common position amongst those with more personal connection to dementia. Eleanor frames this as a subject‐side telling with ‘I think’ and ‘I don't honestly think’, constructing the talk as belonging to Eleanor's opinion and personal experience and potentially creating more opportunity for her opinion to be challenged than Anita's object‐side construction (Potter 2020). In Anita's account there is no hedging, and her talk is also presented as objective fact (Edwards 2007). The talk of participants who did not have dementia tended to be more hedged, for example, Violet (ex‐carer) says:

I think a healthy lifestyle is good generally, but it won't hurt I suppose to have a good diet and exercise and everything, but I'm not sure it is the magic bullet really.

Violet (ex‐carer)

Violet's dementia preventability discourse contains more hedging: ‘I suppose’, ‘I'm not sure’, but still carries the message that dementia cannot be prevented purely through keeping an active and healthy lifestyle. Instead, keeping active is viewed as something that is generally helpful, but only as being able to delay progression of symptoms, not as preventive. Media messages around risk reduction are therefore presented as inaccurate on both a general and personal level. Participants did discuss non‐modifiable risk factors for dementia (genes such as the APOE4 gene, and that getting dementia is ‘just bad luck’—Michelle, carer) proposing alternative causes for dementia that lie outside individual control (cf. Lawless et al. 2018a). However, Geoff (carer) and Julie (person with dementia) both identify a link between health behaviours/lifestyle choices and causation of vascular dementia:

but of course you can always say that that eating a healthy diet will help because it reduces the risk of perhaps having a stroke and and developing vascular dementia.

Geoff (cares for wife)

I think you can look at risk reductions so that there are some dementias, for example, um:: in, vascular dementia that um:: that for example, health‐ um healthier diets such as Mediterranean diet and um £sorry Anita, but not‐ not smoking£ ((smiles)).

Julie (person with dementia)

Geoff and Julie describe links between health behaviours (diet and smoking), strokes and subsequent vascular dementia. This also demonstrates conflicting ideologies discussed by the same participants. Billig et al. (1988) use the term ideological dilemmas to describe this phenomenon where seemingly contradictory views about the same issue are held within one person. Here the dilemma can be encapsulated as ‘my/my loved one's dementia was unpreventable by changing my/their lifestyle’, versus ‘vascular dementia could be prevented through lifestyle management’. The links between cardiovascular risk factors and vascular dementia are more established than those between other forms of dementia in both public perception (Shepherd et al. 1997) and research (Anstey et al. 2019). This could explain the departure in viewpoint for vascular dementia, and how it is displayed as common knowledge by Geoff, ‘but of course you can always say…’.

Julie's talk specifically refers to Anita, another participant in the group who has a diagnosis of mixed dementia (vascular dementia and Alzheimer's disease) and who has been smoking on screen throughout the focus group. In response (but not shown here), Anita echoes Julie's smile and laughter, and Julie then downgrades her claim to one that is not definitive by saying that a lot of people who smoke do not get dementia. This also links to another potential danger of healthist messaging related to dementia: that there is still so much we do not know for certain about dementia causation, and that risk factors themselves are not causes—‘they are at best health‐related factors whose effects accumulate over time and are not dementia specific’ (Leibing and Schicktanz 2020, 2). Our findings therefore indicate that people diagnosed with vascular dementia may potentially be subject to stigma beyond that of other forms of dementia and this, as also discussed by Peel (2014), appears to align with healthist framings. Future research could explore this finding further by focusing upon the potential impacts of media representations surrounding vascular dementia with various stakeholders affected specifically by it, which leads into the second part of this theme on the perceived impacts of dementia media representations.

#### 3.1.2 Dementia as Preventable

In the second part of the dementia preventability theme, participants described and discussed the implications of media representations of dementia risk. All participants viewed discourses around risk reduction in the media as ‘simplistic’, particularly in relation to some newspaper headlines implying that doing (or not doing) one specific thing will reduce dementia risk, echoing findings of Petersen and Schicktanz (2021). Again, those with more personal connection to dementia discussed the impacts of these messages as placing blame for their condition on people who have dementia, without being prompted about this possible impact by the facilitator.^2^

Anita (person with dementia) talks about the excerpt from Allen (2021) as ‘patronising’ and carrying the message that ‘it's up to you to prevent Alzheimer's’, implying the article's messaging is healthist. This encapsulates the consensus of participants personally affected by dementia, that media coverage of dementia risk research incites blame and individual responsibility for dementia through implying dementia comes about by not exercising your brain and body. Participants did not explicitly use the term ‘healthism’ but alluded to key components of it through terms such as ‘victim blaming’ (Henry, academic), ‘fault’ (Eleanor, person with dementia, Hannah, ex‐carer and academic), ‘blame culture’ and ‘responsibility’ (both Julie, person with dementia). For Henry (an academic with less personal connection to dementia), however, this media discourse is interpreted as something which may be comforting for people who do not have dementia:

it's slightly more comfortable to think that people‐ if‐ you get ill when you've done wrong or failed to do right [three lines omitted] £but it doesn't always apply£ and all too often it's it's purely random, the randomness of genes and the randomness of particular environmental exposures: and things you have no control over um but, but that's scarier to think about than, than, than some kind of cause that you have some control over.

Henry (academic)

Henry provides a point of view for how people not affected by dementia may view media discourses about reducing individual dementia risk, positing a hypothetical moral relationship between health and behaviour: that ill‐health comes about by ‘doing wrong or failing to do right’. This identifies healthist discourses around dementia risk messaging in the media: it is not enough to have not done anything ‘wrong’ such as smoking, or having an ‘unhealthy’ diet, you must also work hard to avoid risk by doing all the ‘right’ things (see also: Cheek 2008; Marhánková 2022). Henry presents this discourse as something which exists in the world, an object‐side telling, rather than something he personally agrees with. He contrasts this point of view with another object‐side construction: that causation is random and predetermined due to ‘things you have no control over’, such as environmental exposures and genetics. This is suggested to be ‘scarier’ than discourses which treat humans as having control, and therefore responsibility, over what happens to their health. Henry's talk therefore echoes Crawford's (1977) writing on victim blaming ideology which also creates a ‘politics of diversion’ by downplaying or ignoring evidence of environmental impacts on health and knowledge of human behaviour. Henry's mention of ‘environmental exposures’ is notably the only mention of risk factors outside of an individual level of control in all the focus groups, although this may be less surprising given it was not something that was directly asked about. From a healthist perspective, Crawford (1977, 672) argues that complaints about environmental hazards can be reframed as ‘over‐indulgence from the good society’ that must be curbed. The general absence of discussion of societal‐level modifiable risk factors here could reflect the largely individual and healthist framing of dementia media representations (see Daube et al. 2023).

Healthist representations of dementia risk imply that people with dementia did not exercise restraint or failed to be motivated to maintain a healthy lifestyle. This can be extrapolated to imply something about people with dementia personally, as part of their identity, promoting a homogenising view of them as lazy, unmotivated and deserving of their diagnosis, placing blame at the feet of the individual (Peel 2014; Wilson and Anstey 2024). Therefore, whilst media representations of ‘individual’ risk factors may lead those currently unaffected by dementia to change their behaviours, or provide comfort through the illusion of control, for people already affected they can be detrimental (Peel 2014). This healthist viewpoint labels people with dementia as ‘unhealthy’—since for people to claim a ‘healthy’ self, there must also be the creation of ‘unhealthy’ others (Crawford 1994). Henry's words above are a clear illustration of this process (‘it's slightly more comfortable to think that people‐if‐ you get ill when you've done wrong or failed to do right’), where a distinction can be created between ‘us’ and ‘them’, the ‘ill’ and the ‘well’. Even though Crawford did not explicitly discuss dementia in relation to healthism, we can observe how the viewpoints on health as a moral reward for doing right/not doing wrong are pervasive not only in dementia media representations but also in the talk of participants with less personal connection to dementia.

### 3.2 Demonstrating Dementia Diligence

Participants all demonstrated an interest in continually keeping up to date with dementia news. They presented themselves as people who critically and selectively engage with information from the media generally and dementia news more specifically, and who also turn to alternative sources of information. Media information about dementia was generally regarded as untrustworthy and sometimes contrary. Newspaper headlines were commonly presented as exaggerating or oversimplifying research findings with participants using phrases such as ‘sensationalised’ (Emma, GP; Gemma, ex‐carer and OT), ‘eye‐grabbing headlines’ (Julie, person with dementia), and ‘headline seeking’ (Geoff, carer) to describe this. This finding is in line with research by Petersen and Schicktanz (2021) who examined German media, nursing and medical science discourses on dementia, finding that information on individual risk factors is often oversimplified, specifically in media and nursing science discourses. Participants further displayed media as untrustworthy through discussing a need for ‘fact checking’:

If I needed to burrow down into something more specific I would check out the credentials of it and if I wasn’t familiar with it I would do a bit of googling, cos I know it’s so e‐ easy to get‐ y’know confident sounding but £rubbish information if you’re not careful£.

Henry (academic)

The notion that it is easy to be misled by information in the media results in participants describing a responsibility to fact check the information they consume. Henry posits an individual responsibility for doing so in that ‘if you're not careful’ you can be misled. This was also echoed by people with closer personal experience of dementia in needing to ‘read between the lines’ (Julie, person with dementia) and ‘go deeper’ (Hannah, ex‐carer and academic) so as not to be wrongly or selectively informed. By describing these processes participants present themselves as diligent health subjects. In so doing, rather than calling for media outlets to increase the depth or quality of their reporting, they instead reinforce the role of individual responsibility. Participants also commonly referenced the source of their news as separate from the media—such as from research (e.g., Martin, person with dementia; Geoff, carer) and from people with lived experience (Gerry, carer; Gemma, ex‐carer and OT).

The participant diligence reported here demonstrates the potentially far‐reaching nature of healthism: that it is not only a responsibility for health behaviours, but also for the health information that you take on board, therefore contributing to an ever‐vigilance (Cheek 2008). Indeed, Hannah (ex‐carer and academic) specifically used this term, stating ‘there's so much vigilance that comes into the news and information’ when framing her perception of individual responsibility. Here, there are echoes of Crawford's (2006: 415) suggestion that health consciousness and information have become increasingly unavoidable and that the constant sharing of medical research beyond the medical community ‘demands close attention by consumers of health information’. Crawford (2006) proposes this also contributes to a contradiction whereby the constant flow of health information, combined with societal pressure to maintain health, can ironically increase anxiety rather than provide reassurance. As Geoff (carer) put it, ‘the more you read, the more frightened you become’, and in the carers' group especially, participants advocated for actively curating the sources that you do and do not read to prevent these increases in fear.

There are also implications here for who or what is treated as a trustworthy source. Where all participants may draw upon narratives from academic sources, mainstream media, charities and personal experience sources such as friends or groups via social media platforms such as Facebook, people with dementia and carers were more explicit about the information they take on board needing to be informed by people with lived experience, regardless of the source. This is exemplified by the talk from Eleanor below:

It's very hard to trust, the vast majority of it really. Uh:m you know, unless it is something that is::, uh you know, there's people with dementia who are sort of involved in it and it's people speaking from first‐hand experience?

Eleanor (person with dementia)

Furthermore, highlighting the importance of the grounding of information in the reality of lived experience, Eleanor's talk here about the ‘vast majority’ of published information being hard to trust perhaps reflects the continuing underrepresentation of people affected by dementia in mainstream media (Basting 2009; O'Malley et al. 2022). This is even though print media have the capacity to campaign for change and raise awareness around dementia risk factors, and this has been done successfully for professional male footballers who experienced traumatic brain injury from the sport, resulting in subsequent dementia diagnoses (Putland et al. forthcoming).

## 4 Discussion and Conclusions

Participants responded differently to preventive media messages about dementia based on their personal experiences. Carers and people with dementia repeatedly refuted media claims suggesting dementia could be delayed or prevented. In contrast, individuals with less personal connection to dementia viewed these messages as educational and potentially supporting positive behavioural changes, while recognising that these narratives are not helpful for people already affected. Healthist discourses that simplistically suggest preventive measures taken by an individual can delay or prevent dementia were experienced as even more personally inapplicable for participants affected by *young*‐onset dementia. The problematic nature of these discourses was felt to go beyond inapplicability and into blame, with some participants feeling an implication that they or their loved one did not try hard enough to prevent or delay their dementia. Our findings highlight how frames of reference can influence how people interpret dementia representations in print media.

Additionally, we have demonstrated how healthist discourses in dementia media messaging can reinforce both public‐stigma and self‐stigma by framing prevention as a matter of individual responsibility. Healthism, as conceptualised by Crawford (1977), positions health as a moral obligation and a personal achievement, often overlooking the broader structural, social, and political determinants of health. When dementia prevention is portrayed through this lens, it implicitly suggests that individuals who develop dementia (especially those with young‐onset or vascular dementia) have failed in their personal health responsibilities. This framing not only marginalises those already affected but also contributes to public‐stigma, by reinforcing societal perceptions that dementia is avoidable through the ‘right’ lifestyle choices. In turn, these messages can lead to self‐stigma, as individuals internalise blame for their diagnosis, believing they are personally at fault for not having done enough to prevent it. T. Nguyen and Li (2020) highlight how such internalised stigma can negatively impact self‐esteem and mental health. Crawford (1977) argued for a more holistic approach to health education that recognises the influence of social and political contexts rather than focusing solely on individual behaviour change. Simply offering more support and preventive services (e.g., brain health clinics) or promoting individualised prevention strategies is insufficient to bring about meaningful, large‐scale changes. This point is echoed in the conclusions of dementia research by Daly (2023) and Walsh et al. (2022), who call for public health messaging and policy that addresses systemic inequalities and supports inclusive, stigma‐reducing narratives. Without this shift, healthist messaging risks perpetuating exclusion and deepening the stigma experienced by those affected by dementia.

Although our study adds valuable information to the understanding of how discourses of dementia risk are received and contested, there are some limitations. No individuals with later stage dementia were included as direct participants, though carers sometimes reported experiences of this group second hand. In addition, although it was not part of our intentional sampling strategy, all participants living with dementia had forms of young‐onset dementia. This may be due to recruitment having taken place online (Twitter, Facebook support groups, email), which may have been less accessible to older people (Age UK 2021). Consequently, voices of older people with dementia and those with more advanced dementia are not represented directly in our research. Future research should aim to recruit a broader age range and stage of dementia, embedding inclusive practices such as those discussed by Smith and Phillipson (2021). Additionally, research could examine whether dementia represents a specific case within prevention discourse, or whether healthism is equally visible in media representations of other conditions such as specific cancers, cardiovascular diseases or mental health conditions.

Crawford's (1994) identification of the 'othering' of 'unhealthy' individuals and the 'internal othering' of those with conditions impacting their identity is particularly evident in dementia discourse, which is often portrayed as eroding personal identity (e.g., Mitchell et al. 2013). Although our study did not comprehensively analyse healthist messaging in political spheres around dementia prevention (e.g., Department of Health 2015; Office for Health Improvements and Disparities 2022), our thematic discourse analysis reveals similar patterns of individual responsibility in print media, which are felt personally by those impacted and exacerbate stigma.

Our findings here concur with those from previous UK‐based studies (e.g., Latimer 2018; Putland and Brookes 2024) and beyond (e.g., Lawless et al. 2018b; Low and Purwaningrum 2020); this raises the question of how to change media discourses to improve dementia risk factor awareness without the oversimplification of straightforwardly healthist messaging that leads to blame (Petersen and Schicktanz 2021). Although dementia charities provide media/language guidance and recommendations (Alzheimer's Society 2024; DEEP 2014), uptake of these appears limited, and even if they were exhaustively followed, it is likely that media outputs could still have some negative impacts. Therefore, fostering inclusive, non‐individualising discourses that move away from healthist framings may require broader, top‐down, structural change. Recent public health initiatives such as the Prime Minister's challenge on dementia 2020 (Department of Health 2015) focus on individual lifestyle risks, overlooking social determinants of health. Within such reports there is an emphasis on financial cost: ‘Dementia takes a huge toll on our health and social care services… predicted costs likely to treble to over £350 billion’ (Department of Health 2015, 3); this frames people with dementia as economic burdens, rather than prioritising equitable access to health education and healthcare. This is a particularly problematic framing given the strong link between low socioeconomic status and dementia risk (Livingston et al. 2020). This focus on cost also perpetuates an idea that ‘we cannot afford dementia’, which can further contribute to stigmatisation (Latimer 2018, 840). Deepening research collaboration with policymakers may strengthen embedding social determinants of health (e.g., education, income, housing, and access to care) into dementia risk approaches. A more inclusive approach, with less dependence on healthist ideals, may not only have impacts for dementia incidence, but could also reduce other related conditions, whilst promoting a national understanding that health is shaped by both individual *and* societal factors. Over time, this could iteratively reshape public discourses.

Ultimately, a more balanced approach is needed in print and wider media communication and within public health policy on dementia risk that acknowledges the complexities of dementia within broader social and political contexts. Focusing solely on individual‐level interventions will not only perpetuate blame and discourage help‐seeking but will also prove largely ineffective at reducing overall dementia prevalence (Crawford 1977; Daly 2023; Walsh et al. 2022). Our analysis shows that Crawford's healthism remains a powerful lens for critiquing contemporary health representations and is increasingly relevant to dementia discourse. By envisioning and advocating for more inclusive, holistic public health communication, we can challenge stigma and support those living with dementia more effectively.

## Author Contributions

**Felicity Slocombe:** writing – original draft, writing – review and editing, project administration, methodology, investigation, formal analysis, conceptualization. **Elizabeth Peel:** writing – review and editing, supervision, methodology, funding acquisition, conceptualization. **Alison Pilnick:** writing – review and editing, supervision, methodology, funding acquisition, conceptualization. **Saul Albert:** writing – review and editing, supervision, methodology.

## Conflicts of Interest

The authors declare no conflicts of interest.

## Supporting information


Supporting Information S1

## Data Availability

The data that support the findings of this study are available on request from the corresponding author. The data are not publicly available due to privacy or ethical restrictions.

## Endnotes

^1^
The 14 risk factors are: less education, hearing loss, high LDL cholesterol, depression, traumatic brain injury, physical inactivity, diabetes, smoking, hypertension, obesity, excessive alcohol, social isolation, air pollution and visual loss (Livingston et al. 2024).

^2^
In the less personal connection to dementia focus group, Henry (academic) said that articles such as Allen's (2021) might convey a ‘use it or lose it’ message. However, when specifically asked if the article blamed people for developing dementia, Henry disagreed, stating that the excerpt shown did not imply this. The other two participants in this focus group displayed agreement with Henry's assessment.
