# Supplementary material for: Providing Hope or Assigning Blame? Healthism in Print Media Portrayals of Dementia Risk and Responsibility
Source: Sociol Health Illn. 2025 Nov 11;47(8):e70115. doi: 10.1111/1467-9566.70115 (PMC12603681; doi:10.1111/1467-9566.70115)
Supplement: Supplementary file 1 — Supporting Information S1 [file SHIL-47-0-s001.docx]

## **Appendix A: Focus group questions and materials**

The below was posted to all focus group participants in advance of our first meeting so that participants could familiarise themselves with the questions if they wanted to and also so that they could ask any queries about the questions during the initial meeting.

**Dementia in the media focus group**

**Hello, thank you so much for taking part in a focus group! Below is information on how the focus group will run, as well as the questions we will cover. On pages 3 and 4 are the newspaper articles we will discuss. I am giving the questions to you now so that you know what to expect for the focus group and can, if you wish, think about what you would like to say.**

Overview

**Guidelines**

- Please find a quiet, private space where other people won’t be visible or audible. This is because I only have permission to record you and not anyone else that may be around you.
- Please have your camera and microphone turned on.
- Please listen to others and don’t speak over people.
- Try to speak one at a time please so everyone can be heard.
- I will try to make sure everyone gets their say but if you feel you want to draw attention please put up your hand.

**Important things to note**

- There are no right or wrong answers – I am interested in your views – I know that people have different views, and this group will involve discussion – but please allow others to speak and respect other people’s opinions.
- Anything you do say will be kept anonymous (unless you have indicated that you consent to being identifiable).
- **All meetings will be recorded** to provide an accurate account of what is said.
- I am here to make sure the discussion stays on topic and that everyone has a chance to express their experiences and opinions. You are the experts in your experience.

**Beginning the meeting**

- I will recap the topic area and purpose of the research.
- I will ask if there are questions at this point.
- **Introductions:** Briefly tell the group your first name, and why you wanted to participate in this research.
- We will then begin the focus group.

Questions

1. What’s the most important source of information for you? (E.g., TV, social media, newspapers)
   1. Why is that? (e.g., do you trust it more, find it most accessible?)

**Questions for each headline/quote (on the other pages) – 2 examples to discuss:**

1. What are your first thoughts about this headline/quote?
   1. How do you think the headline/quote presents dementia?
2. Do you think this media message might impact how dementia is viewed?

****We will have a 10 minute comfort break here****

**We can also take a break whenever you need to.**

**Overall media messaging impacts:**

1. What are the possible implications of these media messages for how people living with dementia are treated by others?
2. Do you think media messages impact concern about developing dementia?
3. Do media messages impact or change **your** behaviour?
4. Do you believe you could reduce risk of developing dementia through making changes to your diet and lifestyle?

Closing

There will be the opportunity to add any further thoughts and experiences around the topic of dementia in the media.

I will email you a webpage with information on dementia organisations and charities for further information or support.

A summary of findings will be presented as part of the free blog writing workshop which you are all invited to attend. You will then be able to publish blogs on our website. Alternatively, the summary of findings can be emailed to you.

**Thank you so much!**

Facilitator name and email address

**Article 1 – from the Mail Online**


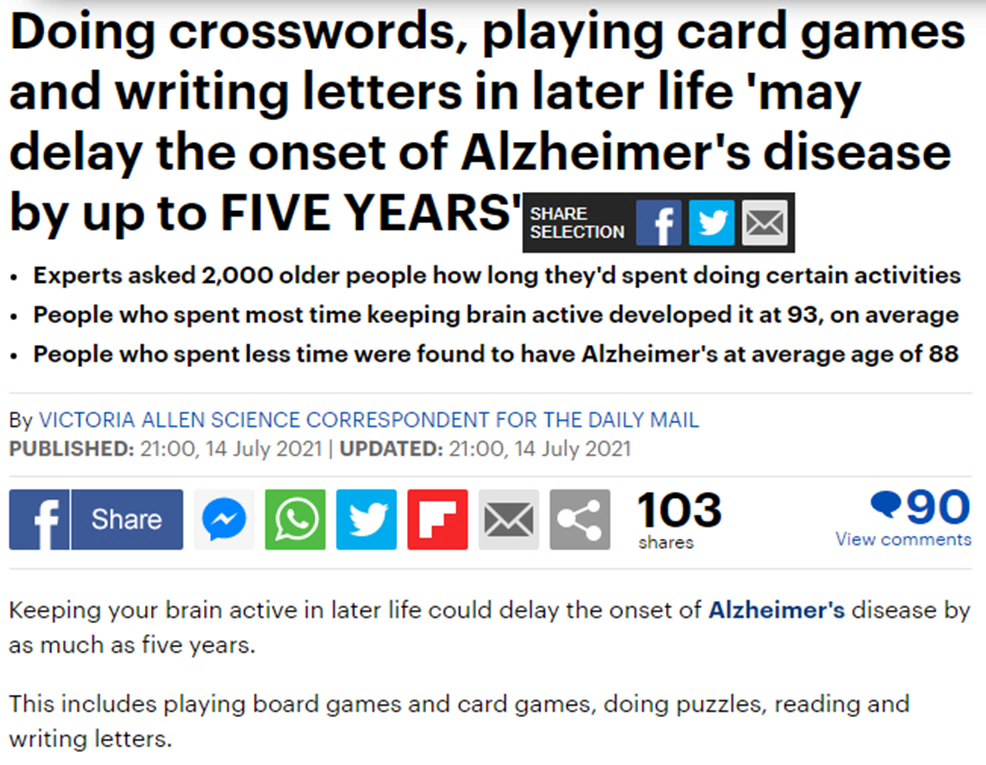


**Article 2 – from The Telegraph**


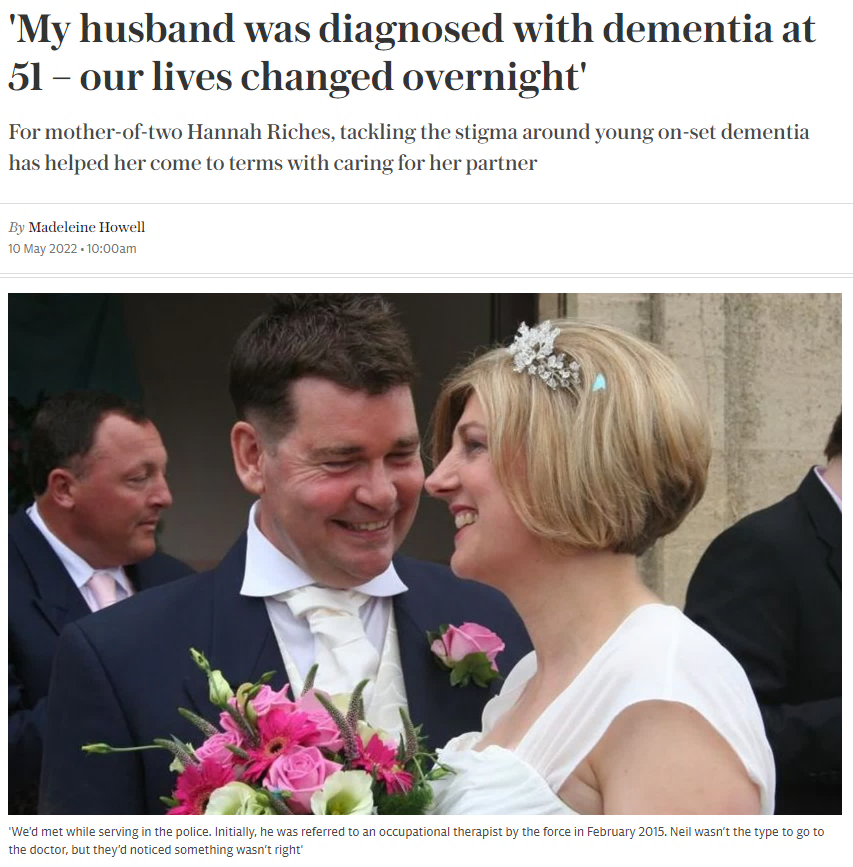


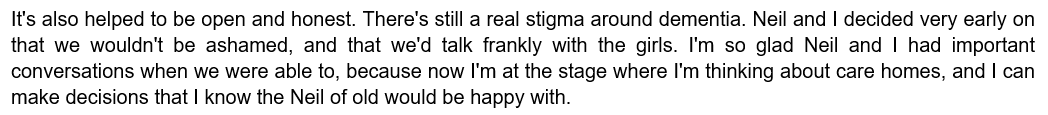
Below is an Extract from the article. For context, when she refers to ‘the girls’ she is talking about their 2 daughters.

## **Appendix B: Selection of newspaper articles for focus group discussion**

Selection of the media examples from newspapers was purposefully done in selecting articles where the content was not distressing, for example stories about abuse and neglect of people living with dementia were purposely not selected. The two media examples came from two different UK national newspapers – one tabloid (Allen, 2021) and one broadsheet (Howell, 2022). We chose the examples via a media analysis of UK national newspapers. We chose Article 1 because it represented a common theme in newspaper reporting on dementia (preventing/delaying dementia through modifiable risk factors) and Article 2 represented a less covered topic (stories from lived experience, specifically of young-onset dementia).
